# Understanding the 'four directions of travel': qualitative research into the factors affecting recruitment and retention of doctors in rural Vietnam

**DOI:** 10.1186/1478-4491-9-20

**Published:** 2011-08-17

**Authors:** Sophie Witter, Bui Thi Thu Ha, Bakhuti Shengalia, Marko Vujicic

**Affiliations:** 1 Health Portfolio, Oxford Policy Management, 6 St Aldate's Courtyard, 38 St Aldates, Oxford OX1 1BN, United Kingdom of Great Britain and Northern Ireland; 2 Hanoi School of Public Health, 138 Giang Vo, Ba Dinh, Hanoi, Vietnam; 3 Human Development Network, The World Bank, 1818 H St NW Washington, 20433 USA

## Abstract

**Background:**

Motivation and retention of health workers, particularly in rural areas, is a question of considerable interest to policy-makers internationally. Many countries, including Vietnam, are debating the right mix of interventions to motivate doctors in particular to work in remote areas. The objective of this study was to understand the dynamics of the health labour market in Vietnam, and what might encourage doctors to accept posts and remain in-post in rural areas.

**Methods:**

This study forms part of a labour market survey which was conducted in Vietnam in November 2009 to February 2010. The study had three stages. This article describes the findings of the first stage - the qualitative research and literature review, which fed into the design of a structured survey (second stage) and contingent valuation (third stage). For the qualitative research, three tools were used - key informant interviews at national and provincial level (6 respondents); in-depth interviews of doctors at district and commune levels (11 respondents); and focus group discussions with medical students (15 participants).

**Results:**

The study reports on the perception of the problem by national level stakeholders; the motivation for joining the profession by doctors; their views on the different factors affecting their willingness to work in rural areas (including different income streams, working conditions, workload, equipment, support and supervision, relationships with colleagues, career development, training, and living conditions). It presents findings on their overall satisfaction, their ranking of different attributes, and willingness to accept different kinds of work. Finally, it discusses recent and possible policy interventions to address the distribution problem.

**Conclusions:**

Four typical 'directions of travel' are identified for Vietnamese doctors - from lower to higher levels of the system, from rural to urban areas, from preventive to curative health and from public to private practice. Substantial differences in income from formal and informal sources all reinforce these preferences. While non-financial attributes are also important for Vietnamese doctors, the scale of the difference of opportunities presents a considerable policy challenge. Significant salary increases for doctors in hard-to-staff areas are likely to have some impact. However, addressing the differentials is likely to require broader market reforms and regulatory measures.

## Background

Motivation and retention of health workers, particularly in rural areas, is a question of considerable interest to policy makers internationally. It is widely accepted that a key constraint to achieving the MDGs is the absence of a properly trained and motivated workforce and improving the retention of health workers is critical for health system performance [1]. Increasing attention is being paid to understanding the labour market dynamic in health [2].

A systematic review of studies on motivation and retention identified seven major themes: financial incentives; career development; continuing education; facility infrastructure; resource availability; management factors; and personal appreciation [3]. The review concluded that while motivational factors are undoubtedly country specific, financial incentives, career development and management issues are core factors. Nevertheless, financial incentives alone are not enough to motivate health workers. The review finds that recognition is highly influential in health worker motivation and that adequate resources and appropriate infrastructure can improve morale [3]. Internationally, there is still considerable debate about the right mix of interventions to address shortages caused by internal and international migration, both for doctors and other types of health workers.

The overall supply of health workers in Vietnam (0.56 doctors per 1000 population, 0.77 nurses and 0.3 pharmacists) is close to the Southeast Asian average but below the regional averages for Western Asia. In comparison with the Africa region, it has more than twice as many doctors per person and five times as many pharmacists, but fewer nurses [4]. The main challenge is the distribution of health staff. Its urban population accounts for 27% of total national population but the majority of university pharmacists (82%), doctors (59%), and nurses (55%) work in urban areas [5]. Remote areas - such as the Northern Uplands provinces or Central Highlands - have fewer health workers per capita, relative to Ministry of Health (MOH) staffing norms, and relative to funded positions. In Lai Chau province, for example, only 3% of community health stations have a doctor, while in Dien Bien the proportion is 16%, 22% in Son La and 24% in Cao Bang (all remote provinces). The shortage is also severe for highly skilled cadres and district level facilities. For example, only 23% of medical staff are graduates in the Central North coastal area (the rest having secondary education or less).

Understanding the labour market dynamics which lead to this distributional challenge was the focus of this study. There is at present very little published (at least in English language journals) on the factors affecting the willingness of medical doctors to accept and remain in posts in rural areas of Vietnam.

This study forms part of a labour market survey which was conducted in Vietnam in November 2009 to February 2010. The objective of the overall study was to understand the dynamics of the health labour market, how doctors make choices between postings and what might encourage them to remain in post in rural areas.

## Methods

The study had three stages: the first used qualitative techniques and a literature review (of English-language sources) to probe doctors' willingness to work in rural areas and the factors that might improve retention. The second involved a structured survey to establish doctors' characteristics. The third used contingent valuation to establish the responses of doctors to changed job attributes. This article describes the findings of the first stage - the qualitative research which fed into the design of the questionnaire and contingent valuation.

The focus of the study in Vietnam was doctors, as this is the main cadre of health worker providing clinical and preventive care, and the one with the greatest overall shortages and imbalances between remote and urban areas. The master plan for human resources envisages a ratio of 8 doctors per 10 000 people, while in 2008 the level was 6.5 [5]. In addition, 60% of doctors work at national or provincial level.

Three tools were developed and piloted: a set of topics for key informant (KI) interviews at national and provincial level; an in-depth interview guide for doctors; and a guide for focus group discussions to be used with medical students. The more sensitive nature of the discussions on pay meant that a focus group approach was not deemed appropriate for serving doctors.

The questions for policy-makers focussed on problem identification, their perception of meaningful attributes for health staff, and the policy options under consideration to address the problem. The question guide for the in-depth interviews with doctors focussed on career choices and routes, the desirability of different job attributes and their priorities for change. Finally, the guide for medical students was focussed on their motivation and expectations of the profession, their willingness to accept different kinds of work, and what factors would motivate them to take work in rural areas.

**Figure 1:**
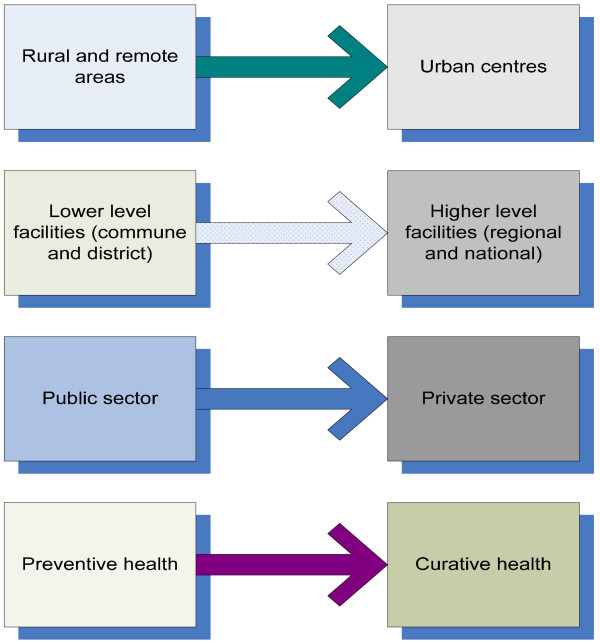
**The four typical directions of movement for Vietnamese doctors**.

Sampling was based around seeking to capture views relating to the four main directions of internal migration in the Vietnamese health market, as suggested by initial discussions with national key informants (see Figure 1).

In practice, these four 'directions of travel' are linked. Almost all high-level public health facilities are located in the cities, while low level ones are located in the rural areas (districts and communes). Private facilities are also clustered in urban areas, while preventive work is carried out primarily in the public sector.

The participants in the qualitative research are summarised in Table 1.

**Table 1:** Outline of participants for qualitative research

Type of respondent	Workplace	Themes	Tool	Number	Total
Policy-makers	National level, Ministry, development partners	Perception of problem; views on drivers for MDs; what policy options are realistic	Key informant interviews	4	4

Health managers	Provincial health department	Overview of HRH in the provinces, with emphasis on the rural and remote areas	Key informant interviews	1 × 2 provinces	2

Doctors	In remote areas and at lower levels (district preventive department, district hospital and community health centres)	Understanding the reasons why they stay and work in these places	In-depth interviews	3 × 2 provinces	6

Doctors	Leaving district level to work at provincial level (preventive sector, curative and private sector)	Understanding the reasons why they left their former workplaces to work in new posts; investigate what might bring them back	In-depth interviews	3 × 2 provinces	5

Medical students	Medical universities	Understanding their expectations and intentions regarding future employment	Focus group discussions	1 FGD × 2 universities (6-10 participants per group)	15

In addition to the national level, the exploratory research took place in two provinces: Lao Cai and Thai Binh. The former was chosen as representing a highland province with low density of doctors, and difficulties of retention linked to a remote and difficult location. Thai Binh is in the Red River region surrounding Hanoi. The main challenge in this area is the pull of lucrative employment in the capital city.

All 32 participants were chosen purposively. Key informants at national and provincial level were chosen on the basis of their posts. In each province, three doctors with more than five years of working experience at district hospital, district center of preventive medicine and commune health center, respectively, were selected. For the other 3 doctors, one was chosen who had moved from preventive to curative care, one who had moved from lower to higher levels (commune or district to provincial or central level) and one who had shifted from the public to the private sector.

Two groups of final-year medical students were invited to participate in the focus group discussions in two medical schools (Thai Nguyen and Thai Binh). The schools were chosen on the basis that they are not in Hanoi (all students in Hanoi tend to stay there) but, rather, are in areas where the students face a more real choice between going to rural areas and leaving for the cities.

In terms of their characteristics, about half of doctors (6/13) were aged from 30 to 39 years old; some of them (2/13) were 40-49 years old and just one was 50-59 years old. All students were aged from 20 to 29 years. Of the overall sample of 32 participants, 23 were male and 9 female (a bias which is close to the national average for doctors). For the doctors interviewed in-depth, three had undergone regular medical school training, five were upgraded assistant doctors and three had postgraduate qualifications.

It should be noted that in Vietnam regular doctors are recruited through a competitive national entry exam and study full-time for six years at medical universities. Upgraded doctors have started as assistant doctors (with three years study at medical colleges), but after working for some years in the health system can study for four years at medical universities to be upgraded. The entry exams for upgraded doctors are less competitive, and the upgraded doctors therefore have lower status, although they are entitled to carry out similar work to regular doctors. Assistant doctors can only treat common diseases, and generally work at the commune level.

National level interviews were conducted in English by an international researcher. For provincial level and below, interviews and focus groups were undertaken by a senior researcher from the Hanoi School of Public Health. The discussions were conducted in privacy to ensure the confidentiality of the work. Written consent was obtained from each participant. Approval from the study was given by the Internal Review Board of the Hanoi School of Public Health.

All in-depth interviews and focus group discussions were digitally recorded and transcribed in Vietnamese. All transcriptions were coded in Nvivo 2.0. About 30 codes were identified during the analysis of data.

## Results

The results are presented thematically, integrating responses from all respondents.

The first section addresses how the problem is perceived at the national level. The next section examines factors that influence recruitment and retention, including income, working conditions, management and supervision, career development and factors linked to living conditions. The third section examines attitudes to working in different locations and roles. Finally, respondents' overall ranking of the different factors is described, and current policy initiatives in relation to rural retention examined.

### 1. Problem analysis

Overall, the problem of distribution of doctors is seen as real but not acute by national level KI. Key informants all agreed that the community and district facilities face the greatest shortage of doctors in absolute terms. There is a vicious circle in relation to utilization: utilization is lowest at lower levels because of lack of confidence in the quality of care and equipment levels, which means that it is harder to justify higher-level human resources when patients are by-passing to the higher levels. The provinces have some problems but these are less severe. On the other hand, there is an 'artificial shortage' of doctors in big hospitals due to overload of work (many diseases can be treated at lower levels but patients are still referred to higher levels).

The communes often use assistant doctors, who can upgrade to doctor status with a four-year training course. They do not have the skills necessary to work in hospitals and so are unable to move away from the community level. However, when district hospitals have shortages of staff, they may use these upgraded assistant doctors. Most of the doctors at commune level are upgraded assistant doctors.

### 2. Factors affecting recruitment and retention of doctors in rural areas

This section presents the findings of the interviews, following the themes laid out in Table 2.

**Table 2:** Topics for in-depth interviews

Motivation to join the profession	
**Domain**	**Topics**

Remuneration	• Salary levels
	• Other public remuneration - allowances, etc.
	• Ability to combine with private practice

Working conditions	• Availability of equipment
	• Working conditions
	• Workload

Non-financial rewards & career development	• Support and supervision
	• Social relationships
	• Career development
	• Access to training

Wider environment	• Housing
	• Education for children
	• Living conditions in the area generally (transport, amenities etc.)

#### Motivation to join the profession

Medicine is a high-ranking profession traditionally in Vietnam and this factor - social recognition and respect - was cited as the foremost reason for joining the profession by medical doctors.

"Other professions might be better than the medical profession in terms of money, but social respect is lower than for the medical profession. For example, when a patient is saying "Greeting doctor", this is very respectful and we feel very proud about this."

- (Medical doctor)

#### Salaries and remuneration

One of the main challenges for retaining doctors in rural areas is the multiplicity of sources of revenue for doctors in Vietnam, most of which favour the high-level facilities and urban areas. There are at least eight main channels of pay and material benefits, which are discussed in turn.

##### (1) Salaries

The scale of government salaries is standard for all doctors. The starting salary level of a doctor is 1.5 million Vietnamese Dong (VND), about US$ 77, which is 2.34 times the minimum wage. There is a national pay scale, which rises with seniority (a small increase every three years). One key informant cited 2 million VND per month as an average salary. Pay is set by the Ministry of Finance, together with the Ministry of Labour, Invalids and Social Welfare (MOLISA).

The doctors' main reported household expenditures are for housing, food, university and school fees, and other social activities (for example, wedding gifts or funeral expenses). Monthly income only covers about two or three weeks' expenditure. Therefore, people have to undertake additional activities to make up the shortfall. National key informants concur that salaries and allowances are insufficient to live on. Doctors are paid on a par with teachers; this is perceived as wrong, given that they train for almost twice as long (6-7 years).

According to national level informants, even if salaries were doubled they would still be insufficient. At least three times the current levels in rural areas would be required to even out pay to any appreciable degree (key informants pointed to the example of Thailand). Some hospitals in Hanoi that treat government officials have tripled pay levels (presumably to stop staff charging these high-ranking patients). This might indicate the magnitude of increase that is needed to counter informal pay. These estimates were supported, and even augmented, by the lower level interviews (medical students, for example, were unwilling to be posted to rural areas, even if salaries were tripled).

##### (2) Allowances

The government introduced higher allowances for doctors in disadvantaged rural areas (since early 2009 they have received a 70% top-up to salaries) but this is considered too low to have an impact by national level KI. Many other supplements are paid, including for leadership roles, regional supplements, and occupational supplements. Doctors can obtain an occupational supplement of 35% of salary if they work in the preventive sector; 30% if at district level (hospital); or 25% if at the community level. A doctor working at a Commune Health Centre (CHC) will receive a 'dangerous job' allowance, which is small (about US$ 2 per month). Doctors working in CHCs in border areas (this applies to only a few selected CHCs) can receive an additional border allowance of 30% of their salary.

##### (3) Pay for performance

Hospitals pay doctors according to the number of procedures that they carry out (at least, for surgery and other specified procedures). The payments are set by the government and are quite low (about VND 30 000 VND per procedure - almost US$ 1.5 - although this may increase shortly).

There is also pay for performing night duties, depending on the level of facility. Doctors reported receiving an allowance for night duties - up to VND 90 000 VND per night (about US$ 5) at a district hospital and 25 000 VND (US$ 1.3) per day at community level.

##### (4) Profit-share

Under the hospital autonomy regulation (Decree 43), facilities can set aside part of profits for staff bonuses. The decree states that profit-share cannot be more than three times total payroll. The bonuses are meant to reward productivity but, typically, are shared out using a standard formula that does not reflect actual activity. The bonuses are not openly disclosed, however.

Financial autonomy does not apply to communes. Generally, the higher the level of facility, the higher the profit-share. Posts at central hospitals are very lucrative: doctors are reported to buy their posts from managers.

Rather than increasing basic pay, the government has allowed profit-sharing to increase to fulfil aspirations. The problem is that these depend on local ability to pay, which is obviously lower in rural areas. One key informant estimated that urban hospitals add about 200% to salaries, while rural facilities might only be able to afford 30-50%.

The growing health insurance system also plays a role, according to national key informants. It does not make payments direct to doctors: it pays facilities for drugs and services provided to its members. However, it does not have contracts with community-level facilities in all areas, which again encourages members to seek care at higher levels. District health insurance funds are used to pay provincial facilities for referred patients, who end up using up a high proportion of the monies: doctors at district level are, however, limited in the treatments they can offer, and feel disempowered. Health insurance is exacerbating the problem of by-passing, with higher level facilities overfilled and lower levels under-utilized.

##### (5) Private practice

Private practice outside working hours is legal, and dual or triple practice is the norm, especially in the cities. The main form of private practice is running small private clinics, either at home or in a shared private facility, usually from 5 pm to 8 pm. National key informants indicated that doctors sometimes divert their public patients to their private clinics, either through poor quality during the daytime or by operating long queues.

Under a new law, in draft at the time of writing, public doctors will not be permitted to own private clinics. However, they will still be able to manage them or work there.

Posts in the public sector provide the credibility needed for a doctor to set up in private practice. It gives them a higher reputation and also allows them to refer patients to hospitals more quickly, if needed.

Some hospitals are also reported to operate private clinics within their grounds, which offer elective services at weekends and evenings. These form separate accounting units - how their revenues are managed is not clear or transparent. Working on foreign-funded projects, especially in Hanoi, forms another source of private income.

Private practice is not well regulated: doctors can practice without a licence in some places

Private practice is mushrooming in large cities such as Hanoi and Ho Chi Minh City, while it is still very primitive at the lower levels and almost non-existent in the highland areas. In Thai Binh, there is only one private hospital, while there is no private hospital in Lao Cai. Doctors working in Thai Binh said that they could earn income from private practice. However, doctors working in highland provinces such as Lao Cai, where people are too poor to pay for private services, need to earn additional incomes from raising chickens, rice harvesting or any other available activities.

At district and community level in Thai Binh province, all doctors confirmed that they have a private practice. However, none of them had private clinics: the doctors either go to the patient's house or patients come to their doctor's house. The fees paid for the service are rather low, due to the low economic status of households in the region: about VND 5-7000 (less than US$ 0.5) - which is about 25% of the level charged in Thai Binh city, and about 5-10% of the level charged in Hanoi. The total income generated from private practice was estimated at VNC 1 million per month at community level (about US$ 52) and VND 2 million per month at district level (about US$ 104).

According to the KIs in Thai Binh province, the doctors working in district public hospitals are unlikely to have a private practice because the workload in the hospital is very heavy.

##### (6) Informal payments from patients

Official fees are regulated and low, so patients know that they need to offer top-up payments to get a good quality service. Even those with health insurance make direct payments to staff. Obstetrics and surgery are thought to be the biggest fields for these 'envelopes'.

There are many tales of unethical practices aimed at extorting patient payments, such as telling patients that their urgent operation will be delayed unless they pay some additional funds, or offering a more-or-less-painful procedure, with pain levels implicitly linked to contributions.

It is said by key informants that medical students start out with an idealistic approach but, after several years, most join in these unethical practices in order to raise their incomes and because they have 'caught' such bad habits.

From the customer point of view, there is also an expectation of paying for performance, either before, during or after the care is delivered. This has become a social norm and is accepted behaviour. When people are unable to give gifts to doctors, this makes them feel uncomfortable.

"Now, if a doctor does not accept the money from a patient, the patient might think that the doctor is a little odd. Furthermore, if you do not accept the money, then the patient will worry"

- (Doctor)

However, the in-depth interviews revealed that almost no patients' gifts were received at the community level due to the low economic status of people using these facilities. A few people express their appreciation - mostly with in-kind contributions when they have the opportunity, such as rice in the harvesting season, or chicken or oranges. At district level, patients' gifts are more likely to occur in certain wards and specialties, such as ENT, dentistry, obstetrics/gynaecology or surgery, with value of VND 20-50 000 (US$ 1-2.5). In other wards, such as internal medicine and the examination department, the giving of gifts is uncommon. According to the respondents, the total payment from patients' gifts is not very high at district level. However, the level of money could be higher at provincial level (VND 50-100 000 VND, or US$ 2.5-50). Patients having surgery might pay gifts of about VND 300 000-1 000 000 per operation (US$ 15-52).

##### (7) Payments from drug retailers and revenues from drug sales

According to national KI, drug retailers visit public sector staff, including pharmacists, to promote their medicines, and staff can be offered a 10-20% commission on the value of the drugs that they prescribe. This is especially the case for internal medicine. This practice used to be open but is now illegal. Nevertheless, it continues.

Those in private practice will receive even more - perhaps 50% of the value of the drug sales. This is often their main source of income (they might not even charge for consultations but base their income on the profit mark-up on drugs instead).

At the community level, drug sales are a main source of revenue for facilities. There is no regulation of prices and very limited supervision of prescribing habits. This creates perverse incentives - for example, facilities might prescribe drugs because they are about to reach their sell-by date rather than because patients need them.

##### (8) Income from private investments in facility equipment or infrastructure

Prices for services are set by the government. However, where there has been private investment in equipment or infrastructure, then the facility can set its own prices (and profit-sharing arrangements with staff). Thus, medical staff can invest in equipment (for example, computed tomography (CT) scans and other diagnostic equipment) and then get a 'rental payment' every time they are used (which, if they are referring patients for tests, leads to a very obvious conflict of interest). This is quite legal. According to national KI, hospital directors cannot invest, but they can get proportion of income from others.

##### (9) In-kind and other benefits

Some communes allocate housing or plots to attract doctors, or a relocation bonus, but this depends on the area - there is no national policy on this.

Some areas used to offer stipends to travel home, but the value of these has fallen and they are not much used now. In general, the old socialist approach of having low pay but paying funds for food, transport, gas, electricity and the like is now considered outmoded. These costs should be covered by rural supplements.

#### Facilities and medical equipment

Good facilities and high-tech equipment are attractive to patients and are therefore one of the determinants of funds raised from patients. According to the doctors, overall facilities and equipment are still inadequate. The field visits show that most of the CHCs in Thai Binh province reached the national standard of six rooms per CHC. However, the situation is worse in highland regions such Lao Cai. Most CHCs lack equipment when compared with MoH norms for their level. Since CHCs are only allowed to perform the diagnosis and treatment of common diseases, not much equipment is allocated to this level. Doctors felt that they are useless without equipment at CHCs and expressed the need to have better and more equipment for their clinical work.

At the district level, the facilities are reported to be adequate. They have sufficient equipment for clinical diagnosis and treatment. This is due partly to National Programme 47 for upgrading district hospitals with government credits, and partly to social mobilization through the hospital. However, compared with higher level (provincial) hospitals, the district level is seen as being inadequate. Again, the situation is worse in highland regions such as Lao Cai. Due to the lack of equipment, doctors cannot confirm their diagnoses and need to refer patients to a higher level health facility. Furthermore, the doctors receive no follow-up on patients referred to higher level facilities, so they do not know whether their diagnosis was correct. This does not help them to improve their performance.

People working in preventive health also indicated that they do not have adequate equipment for their work. For example, in the district centre of preventive medicine, they do not have sufficient equipment to carry out environmental checks, for temperature, humidity, noise and so on.

#### Working conditions

Working conditions are less satisfactory at lower levels, according to the national level KI. This includes many dimensions: in addition to less sophisticated equipment, the doctors often have less ability to practise and extend their skills, less intellectual stimulation, less experienced colleagues, poorer patients and lower utilization of facilities in general.

One concern expressed by the doctors interviewed was that most doctors working at district and community levels are former assistant doctors who had upgraded though training. Upgraded doctors are perceived by regular doctors as not very skilful, so a young, regular doctor does not feel he or she can learn much from them. For the young regular doctor, on the job training is important, so they do not want to go to work in a health facility where their colleagues will be upgraded doctors.

In the highland region, due to long distances and bad road conditions, doctors' work is reported to be very difficult. The travel costs of fieldwork are also very expensive for preventive doctors, which causes further burdens for the already badly paid doctors in that region. The long and difficult roads also mean that clinical work is risky, especially for patients' health. This makes the young doctors unwilling to work there, even at the district hospitals.

#### Workload

National level key informants reported that workload is higher in urban areas in general. At the provincial level, there can be an overload, as they receive all of the referrals and yet often there is only one provincial hospital. At lower levels, workload is unlikely to be a demotivating factor, as facilities are under-utilized. In districts, many staff members might work mornings and then take the afternoon off. At the community level, there are intensive periods associated with campaigns, but then quiet periods at other times.

Doctors agreed that the workload at community level is not very high and medical staff members can be more flexible in organizing their work. Doctors working at a CHC can undertake dual work - curative as well as preventive. However, the work is much harder in the highland regions, due to long distances, bad road conditions and cultural preferences. For example, the doctors might have to go to ethnic minority households in the evenings or at night to help with a birth because the some women from such groups do not want to deliver at a CHC.

The workload is higher at the district level. Doctors working at a district hospital report that they have to work until 18:00 in order to see all of the patients. Sometimes, in the examination wards two doctors have to cover the physical examinations for 100 patients per day. In the highland regions, due to the lack of doctors, one doctor is in charge of 40 patients per day. Beside their clinical work, they also have to deal with administrative work.

Staff members working on preventive services also claim to be busy. They have many field activities, including the supervision of different national health programmes at lower levels, as well as undertaking health education. However, due to lack of equipment and funding, not all activities can be implemented.

Overall, the general view is that the higher the level, the higher the workload for clinical staff, while preventive doctors are seen by clinical staff as having a less onerous workload.

#### Support and supervision

According to national level KI, support and supervision are more limited in rural areas compared with urban areas. Supervision used to be provided in an integrated way by the district health centre, which supervised all administrative staff, the preventive and curative services in the district and the community health services. However, in 2003 it was reformed so that district hospitals, district health offices and preventive services were managed separately. This has reduced the supervision of the community health stations, which falls entirely on the district health office now (without the support of medical staff at the hospital, for example). Since each district has 10-15 communities, the support is spread thin. This policy may now be reversed. Staff members in district preventive services often set up outpatient clinics to generate more revenue, which again means that they have little time to supervise the communes.

The people working at the CHC felt that supportive supervision in the field is helpful, as they can learn how to do better work. In addition to monthly supervision, six-monthly and annual comprehensive supervisions are also conducted. The work plan is the main tool that is used for supervision of doctors working in preventive and curative care. The outputs of the work plan are used for assessment of doctors' performance. Very few sanctions are applied to staff members who have not achieved performance targets. Discussion is the main method for handling these cases. Some complaints were voiced about the quality of management: typically, people with technical skills are promoted, but they do not necessarily have management skills.

A range of non-financial rewards have been tried, according to national key informants - awards, medals, certificates - but they tend not to link to performance but, rather, are allocated according to 'fairness' (the spirit of collective endeavour makes it difficult to identify high performers).

#### Social relationships

Medicine is a high-ranking profession traditionally in Vietnam and this factor - social recognition and respect - was cited as the foremost reason for joining the profession by doctors.

Doctors reported satisfactory relationships with colleagues - they help each other, and there is no discrimination among them. However, the relationship with clients is not always smooth. This was reported in both curative and preventive work. However, tense relations between doctors and clients are more likely to occur in the clinical wards. Sometimes patients who are drunk come to the hospital, abuse and scold the doctors, or beat the doctors if they are not happy with the treatment they have given. This was however acknowledged to be uncommon.

#### Career development

When asked about promotion procedures, the majority of respondents indicated that promotion is quite transparent and democratic. The Ministry of Internal Affairs developed a guide on promotion procedures and all facilities at all levels must follow these procedures. The main criteria for promotion are high technical expertise, management skills, good relationships with colleagues, and sufficient qualifications. The only complaint was about the time taken for higher levels to approve promotions. In rural areas, however, promotion is not always straightforward. In order to be promoted to be head of a CHC, it is necessary to have a good relationship with the local authority.

Not all staff members seek promotion, however - for example, doctors working at a CHC may not want to work at the district centre of preventive medicine. This is because the salary is low in the preventive sector. At the CHC, they can have greater flexibility and can spend more time in private practice.

At present, there is a clearly established hierarchy in the system, according to national key informants - it is not easy to reverse that to make lower level posts more attractive, unless they are made necessary stepping stones to more remunerative postings.

In my opinion, the career path development is very important, because this will link to salary and other income sources. If we sent them to place with a high salary, but no career path development/no professional expertise development, then they will not wish to go there

- (Key informant).

#### Training

Medical training is not expensive but it is hard to obtain a place, as places are limited and the pass mark very high. This means that bonding policies are less effective, according to national KI, as it is easy to repay the fees and avoid the rural duties.

Even if people from rural areas are given preferential treatment in applying for training places (as happens at present - they can pass on a slightly lower score), they still do not tend to return to their rural areas after years of training in the cities.

Higher (e.g. post graduate) training is a mixed blessing - one key informant pointed out that training is not popular as you have to leave home, and are not always paid (or are not paid in full) while you are undertaking the training. While staff might wish to receive a higher level of training, anyone with higher training does not return to work at lower level facilities - all key informants agreed that training is a 'passport out'.

A new law is coming in on accreditation, which requires ongoing training. However, it is not yet clear what this will mean, in terms of content, and who will pay for and provide this (the individual, their facility, or the government). The government could offer to pay for this as part of a rural retention package.

The in-depth interviews suggest that there are not many training opportunities for doctors working at lower levels. Most doctors working in the district hospitals said that they did not have time for study due to their workload and the lack of doctors in the workplace. Those working in highland areas are particularly unlikely to be sent off for study due to shortages of staff. Furthermore, the opportunity costs are rather high, so not many could afford the programme. However, the need for training (short courses, graduate and post-graduate) was clearly reported.

#### Living conditions

The cost of living is lower in the districts, though not for all items (for example, transport costs add to the cost of imported items). Land and houses are cheap; however, this is outweighed by the lack of good schools for children and the isolation from family, the need to spend money travelling out of the area and so on.

Doctors in lowland areas report that housing is available and affordable - in Thai Binh, for example, the cost of hiring a house is around VND 500 000 (about US$ 25) per month. However, the situation is quite different in highland areas such as Lào Cai. Most doctors said that they have to live in the CHC because housing is very scattered in the highland areas. On this basis, being provided with housing could be an important factor in the motivation of doctors working at the lower levels in the highland areas.

Regarding other amenities - such as cafés, karaoke, supermarkets and so on - doctors at district level were quite content. However, the doctors at community level would like their situation to improve. The schools are reported to vary in quality, and travel distances to work can be long, using poor roads.

#### Overall satisfaction with current work and lifestyle

All doctors were asked whether they were satisfied with their current work and lifestyle, and the majority reported that they were satisfied with the current conditions. The main reasons given were stability of their lives, an interesting job, and being close to their home town and families. Stable lives, including work and income, are the most important factor for doctors who have worked for some time in the rural areas. Their demands are not very high. They only need 'three meals per day', 'to undertake the health care for the local communities', and 'stable work and salary'.

### 3. Willingness to work in different locations and jobs

#### Preventive versus curative

Doctors of all ages suggested that the preferred type of work would be to find a post in the highest possible level of hospital. Curative work was seen as having more immediate and more recognised impact. Moreover, preventive work is seen as less skilled and therefore less respected.

"Preventive work is not important, does not require much knowledge, anybody can do the job and nobody is respected... Only students that do not perform well study preventive medicine"

- (Doctor).

Preventive work is considered less arduous, but also considerably less remunerative, as it fails to offer opportunities for private practice and payments from patients.

A supplement of 35% was introduced for doctors working in preventive health. There is no evidence that it has made a significant difference to retention. According to some national level KI, it should be increased to around 100%.

There is now also talk about supplements for people working with 'disadvantaged medical conditions' - for example, Tuberculosis (TB), Human Immunodeficiency Virus (HIV) and leprosy (to boost entrants to public health and preventive health). While there are some target payments for people working in public health programmes, such as the Expanded Programme of Immunisation (EPI) and HIV, other more lucrative forms of revenue are limited here (for example, gifts from patients, payments from drug retailers and so on).

It is particularly hard to recruit doctors into preventive health in the south, where there are so many private clinics. Areas of special shortage are epidemiology, occupational health and school health.

#### Public versus private practice

Of the 13 doctors interviewed in-depth, 12 were working in government health facilities. All of them advised the newly graduated doctors to work in the public sector. According to them, there are several reasons for this. The first is that jobs in the public sector are more secure and more stable than in private practice. They also identified better management and better professional ethics as factors: the lack of quality assurance and the focus on profit in the private sector has an impact on the quality of services, including a tendency to overuse high technology for diagnosis and over-prescribe medicines for patients. Another factor was lower social recognition of doctors working in the private sector compared with those in the public sector. Moreover, doctors working in the public sector have access to other opportunities - such as promotion, training and other sources of income (for example, gifts from patients), while doctors working in the private sector only receive salaries.

Furthermore, the public doctors still can work in the private sector (at a hospital or clinic), so they can do dual practice and obtain dual incomes.

Many students in the last year of medical school felt that the private sector is more demanding than the public sector in terms of working hours and responsibility. Private doctors need to work for 6 days a week, instead of the five-day week of the public sector. Also, they are required to be solely responsible for service delivery, while this is a collective responsibility in the public sector.

Those who had left the public sector did however identify better working conditions in private practice as being an advantage of leaving the public sector.

#### Urban versus rural; higher levels versus lower

The most important reason why doctors preferred to work in urban areas was the better working conditions on offer at national and provincial levels. These include the availability of medical equipment for professional activities and also access to highly skilled professional colleagues working in the same organization. The second reason was the higher incomes that can be earned in urban areas, due to higher salaries and additional sources, such as from a hospital fund and private practice. The third reason was better living conditions - including housing, transport, schooling and social activities such as entertainment, cinemas, cafés and so on. Access to training was also identified as being better in the cities than in rural areas.

### 4. Overall ranking of factors influencing decision to work or stay in rural areas

Key informants at national level felt that inequality of pay was the main factor behind the problem of recruiting and retaining doctors in remote areas, followed by working conditions (poor equipment and so on) and opportunities to learn (the limited range of work and development opportunities at lower levels).

Seven attributes affecting recruitment and retention were highlighted by researchers, based on the most salient factors mentioned in interviews. (The number was limited by the discrete choice experiment into which the research was feeding). The doctors were asked to rank the most important factors from the list that would motivate them to work in rural areas. Each respondent ranked the attributes, with 1 given to the most important attribute and 6 to the least important. (Attributes that were not ranked were given the value 7.) A compound score for each attribute is then obtained by applying a weight to each ranking, with ranking 1 receiving a value of 1 and ranking 7 a value of zero. The weights are linearly decreasing from 1 to 0. All scores are added, so that the maximum possible score for each attribute is 10 and the minimum 0.

The weighted scoring suggests that the order of importance of attributes to encourage doctors to work and stay in rural areas is as follows, in decreasing order of importance:

1. Salary (score: 9.2)

2. Working conditions (score: 7.7)

3. Training Opportunities (score: 5.3)

4. Allowances (score: 5)

5. Career development (score: 4.3)

6. Living conditions (score: 3.7)

7. Supervision and Management (score: 0.8)

Table 3 gives the ranking by different participants.

**Table 3:** Ranking of attributes by participants

Categories	Sex (M/F)	Salaries	Working condition	Training opportunities	Career development	Allowance	Living condition	Supervision & management
**MD at district hospital**								

*Thai Binh*	F	1	2	3	4	5	6	

*Lao Cai*	M	2	1			3		

**MD at district centre for preventive medicine**								

*Thai Binh*	*M*	*1*	*2*	*3*	*4*	*5*	*6*	

*Lao Cai*	*M*	*3*	*2*	*4*	*5*	*1*		*6*

**MD at commune health centre**								

*Thai Binh*	M	1	2	3	4		5	

*Lao Cai*	*M*	*1*	*3*			*2*	*4*	

**MD who had left from public to private**								

*Thai Binh*	M	1	3	4	2	5		

*Lao Cai*	None							

**MD who had left from lower to higher levels**								

*Thai Binh*	*M*	*5*	*3*	*4*	*2*		*1*	*6*

*Lao Cai*	*F*	*1*		*2*		*3*	*4*	

**MD who had left from preventive to curative care**								

*Thai Binh*	*M*	*5*	*3*	*4*	*2*		*1*	*6*

*Lao Cai*	*M*	*1*	*3*	*4*		*2*		*5*

**Final year students at medical schools**								

Thai Binh	2 M and 3 F	1	2	4	5	3	6	

Thai Nguyen	8 M 2 F	3	1	2				

Note: 1 = most important; 6 = least

### 5. Government policy initiatives to address rural retention

The government has introduced a number of measures recently to address the challenge of rural retention. These include:

**• strengthening health facilities in rural areas **- there has been a great deal of investment to upgrade community health centres and district hospitals, especially in terms of equipment and training for staff. They are now reported to be at an acceptable level, according to national KI

**• introducing health insurance at the community and district levels **- this has been done in the past few years, and has led to an increase of 30-50% in patients at these lower levels

**• rotation of staff to lower levels ***- *Decree 1816 aims to encourage staff from higher levels to support lower level facilities by sending each member of staff for a three-month period to work in the provinces. It is a high-level political initiative. It has not been evaluated but anecdotal evidence suggests that people are not staying long and often pay to avoid staying beyond a token period. It is a short-term measure that is intended to increase exposure to rural conditions, and possibly attract those who are public health-minded, but it is not clear whether it is increasing willingness to stay in rural areas

**• incentives for staff in rural areas ***- *there are many local initiatives to offer accommodation, houses, supplements, lump sums for relocation and so on. However, the total picture is not known at the national level. It is possible that this is simply allowing the richer provinces to attract more staff at the expense of poorer provinces.

**• selecting local people for training ***- *there is a lower threshold for people from rural areas in terms of the national examination entry standards for medical school

**• upgrading assistant doctors ***- *young students stay in the cities after training, even if they come from remote areas, so the best approach is to upgrade those assistant doctors working in the remote areas.

In addition, there is discussion of passing a regulation to force newly graduated students to spend one to two years in rural areas. Another option under discussion is to add rural work as a factor favouring promotion.

In relation to salary increases, there is openness to discuss increasing salaries by a significant margin (for example, 200%), according to national KI, but some of these funds would have to come from local sources and the top-ups would need to be targeted on areas with staffing below a certain level (that is, not general to all rural areas). Additional allowances for 'difficult' specialties is also under consideration.

One option would be for the government to increase salaries and outlaw private practice for public workers (as done in Thailand). However, officials fear they would lose too many doctors.

## Discussion

This research is based on a limited number of interviews at national level and in two provinces of northern Vietnam. The results are therefore suggestive, rather than conclusive and may not represent the geographical range of opinions. Conditions in other areas, such as the Mekong Delta, are quite different to the rural and highland north. It is also possible that the small sample contained biases. It was predominantly male, for example, although this does reflect to some extent the gender balance in Vietnamese doctors generally (there were nearly twice as many male doctors as female in 2008, and men are particularly over-represented in hardship areas).

Despite the limitations, a number of important themes have been illuminated, and from a variety of perspectives (policy-makers, managers, doctors and medical students). Where other studies are available, such as [6], they tend to reinforce the conclusions that emerge from this exploratory research. The themes which emerge from the interviews and focus groups map well onto the issues raised by the systematic review on motivation too (financial incentives, career development, continuing education, facility infrastructure, resource availability, management factors and personal appreciation) [3], although, in addition, living conditions and social status emerge as important (and, to a lesser degree, workload).

The views of the three main constituencies - national level key informants, doctors and final year medical students - are largely shared. There are few points of clear disagreement. The policy measures adopted or under discussion by the MoH also demonstrate a shared understanding of the challenges. For example, the programme to improve commune and district facilities responds to the frustrations expressed by participants over poor equipment at these levels. However, the multiple disadvantages at lower levels mean that most regular doctors will still tend to avoid them - hence the focus on developing the cadre of upgraded assistant doctors, who are less likely to be recruited by hospitals.

Although the ranking of attributes might be expected to be very different for the doctors who had stayed working at lower levels and those who had moved to higher levels, private practice and curative care, Table 3 shows that scores were not very divergent across these categories of individual. Salaries were dominant for both groups as first-ranked attribute, though the second-ranked attribute for those who had moved was career development or training, whereas for those who had stayed it was typically working conditions. This perhaps reflects the higher level of ambition of the more mobile group.

For the medical students, those in the lowland areas (Thai Binh) appear to be more financially-oriented in their rankings that those in upland areas (Thai Nguyen).

Doctors who stay at the lower levels tend to have one of two profiles. One group is the general doctors working at district level, who have been there for some time and have family, housing, children and other work which makes them content to stay. The second group is the upgraded doctors, who are required to return to commune or district level to work. Whether they choose to remain based there or are restricted by the lower status given to them is less clear.

The current system, which relies on these 'lower status' doctors to staff the lower level facilities, is a pragmatic response to the preferences of doctors and attraction of the hospitals and urban areas. However, it does reinforce the perception of low-quality care at primary level, and thus contributes to its underutilisation, while hospitals, especially at provincial level, are crowded with patients by-passing the lower level facilities. To reverse this requires effective measures to motivate a mix of doctors to work and stay at the lower levels, at least for some period, while also supporting them with appropriate working and living conditions. Actions to boost the status of preventive health could also be considered, for example by blending preventive work with research activities, or other methods of fostering professional development and peer standing.

One theme which stands out is the complexity of funding sources for doctors in Vietnam, and the extent to which these work against retention at lower levels. Moreover, market reforms and the process of 'privatisation from within' make it hard to equalise pay through salary changes alone - the gaps opened up by the many informal and entrepreneurial channels are simply so large. This is supported by other studies in this field [7]. Market reforms, which were intended in part to offset the problem of low salaries, have increased inequalities, as well as creating a number of problematic incentives, such as supply-induced demand (for high tech equipment and drugs). Some form of regulation (for example, not being able to be licensed as a doctor without undertaking a period of work in rural areas) and market reform appear necessary to limit the supply-induced demand and reduce pay differentials.

Doctors report that the combined official salary and allowance of doctors with more than 20 years work at CHC level will be about VND 3.5 million (about US$ 180) per month, while doctors with similar work experience at a district hospital will receive about VND 6 million (about 310 USD). By comparison, a doctor working in a provincial hospital earns about VND 10 million (about US$ 516) and a doctor working in a Hanoi hospital about 20 million VND (about US$ 1033) from official salaries and allowances. This six-fold difference from bottom to top is magnified by other income sources. Moreover, it emerges that a number of apparently separate attributes, such as equipment, are also linked quite closely to opportunities for the generation of revenue.

On the other hand, it also appears that there is a 'psychological cost' to having to extract surplus from patients, and that there is therefore some scope for a trade-off of lower revenue expectations in return for a higher basic pay, if combined with other professional rewards which emerged as important (such as better working conditions, training opportunities, career development, and improved living conditions). The first three are all seen as feeding into professional development generally, which was highly emphasised by the doctors interviewed here (although again, this is partly linked to future revenue expectations).

According to the National Health Accounts [8], spending on state worker remuneration in 2005 constituted 42% of recurrent spending at state medical facilities and agencies and 32% of total public spending on health. This share is highest at the commune level (62% of recurrent public expenditure on health) and lowest at national (32% of recurrent public expenditure on health). International comparisons indicate that Vietnam has a relatively low share of general government health spending devoted to remuneration, although the average for Southeast Asia is only slightly higher, at 35.5% [5]. There may therefore be scope for an increase in overall public pay. Allowances, for example, currently range from 40-70% of basic salary, while in other countries in the region, allowances are used more seriously to retain staff in rural areas, ranging from 2-5 times basic salary.

Overall, in Vietnam, according to the national health accounts, one third of financing for health care comes from the public sector, which shows the extent to which changes to public pay might be limited, in terms of their ability to counter-balance pay coming directly from the public (through private or public practice). Key informants estimate that doctors in cities earn 80% of their income from private sources and 20% from public sources, compared with doctors in the districts, where the proportions would be reversed.

However, the interviews did not support the idea that private practice is always attractive for doctors - existing doctors recommended that new entrants stay in public services, where they can enjoy security and status without foregoing private revenues. Of the four 'directions of travel', this one was not sustained by the research, because public servants do not need to become private in order to work privately. According to the National Health Survey of 2001-2002, 56% of all private health workers interviewed reported that they were also working in public hospitals or commune health stations. Another study [5] found that 83% of doctors working in state health facilities also worked privately in the community.

## Conclusions

The Vietnamese health system in its current phase presents some particular challenges in relation to maintaining a balance of doctors at different levels of the system. In general, overall numbers are considered to be adequate, but their distribution is unbalanced. Four main types of preference have been identified: for higher over lower levels, for urban over rural, for curative over preventive work, and for public over private practice (although this is largely attributable to their ability to combine the advantages of both while in public employment - 'privatisation from within', in which private revenue streams come to dominate public pay).

In relation to income, a wide array of payment channels were discussed, including salaries, allowances, payments for specific activities, profit-sharing, payments from drug retailers, informal payments from public patients, private practice, inside and outside of the public facilities, and rental of equipment to facilities. Of these, only the first three are within direct public control. The combined income from salary and allowances of a typical doctor at national hospital level may be six times that of a doctor at commune level. All other payment channels reinforce these huge differences.

While doctors emphasised a number of important attributes beyond income which are important to their motivation, most informants placed income first amongst the attributes, and many of the other ones (such as training and equipment levels) are also linked to potential or future income.

The Government of Vietnam is already pursuing a number of policy measures to improve retention in rural areas, which target many of the important factors highlighted by this study, such as working conditions and training. However, these are seen as having had limited effectiveness to date. Significant salary increases for doctors in hard-to-staff areas will have some impact. However, addressing the differentials is likely to require broader market reforms to control some of the exploitative informal charging practices, as well as regulatory measures which mandate a period of rural practice as a necessary step on the path to public employment and promotion.

## Competing interests

The authors declare that they have no competing interests.

## Authors' contributions

SW and BTH designed the qualitative research. SW conducted the key informant interviews. BTH conducted the focus group discussions and in-depth interviews. SW prepared the first draft of this paper. BTH contributed and commented on the draft. BS and MV designed the overall research project and commented on drafts. All authors read and approved the final manuscript.
